# Predicting Enteric Methane Emissions from Dairy and Beef Cattle Using Nutrient Composition and Intake Variables

**DOI:** 10.3390/ani14233452

**Published:** 2024-11-28

**Authors:** Yaodong Wang, Weitao Song, Qian Wang, Fafa Yang, Zhengang Yan

**Affiliations:** College of Information Science and Technology, Gansu Agricultural University, Lanzhou 730070, China

**Keywords:** beef cattle, dairy cattle, methane emission, statistical model, energy

## Abstract

The objective of this study was to develop linear and nonlinear statistical models for predicting enteric methane emissions from beef and dairy cattle (EME, MJ/day). Ration nutrient composition (g/kg), nutrient (kg/day), energy (MJ/day), and energy and organic matter (OM) digestibility (g/kg) were used as predictors of CH_4_ production. Three databases of beef cattle, dairy cattle, and their combinations were developed using 34 published experiments to model EME predictions. Linear and nonlinear regression models were developed using a mixed-model approach to predict CH_4_ production (MJ/day) of individual animals based on feed composition. For the beef cattle database, Equation methane (MJ/d) = 1.6063 (±0.757) + 0.4256 (±0.0745) × DMI + 1.2213 (±0.1715) × NDFI + −0.475 (±0.446) × ADFI had the smallest RMSPE (21.99%), with 83.51% of this coming from random error and a regression bias was 16.49%. For the dairy cattle database, the RMSPE was minimized (15.99%) for methane (MJ/d) = 0.3989 (±1.1073) + 0.8685 (±0.1585) × DMI + 0.6675 (±0.4264) × NDFI, of which 85.11% was from random error and the regression deviation was 14.89%. When the beef and dairy cattle databases were combined, the RMSPE was minimized (24.4%) for methane(MJ/d) = −0.3496 (±0.723) + 0.5941 (±0.0851) × DMI + 1.388 (±0.2203) × NDFI + −0.027 (±0.4223) × ADFI, of which 85.62% was from the random error and the regression bias was 14.38%. Among the nonlinear equations for the three databases, the DMI-based exponential model outperformed the other nonlinear models, but the predictability and goodness of fit of the equations did not improve compared to the linear model. The existing equations overestimate CH_4_ production with low accuracy and precision. Therefore, the equations developed in this study improve the preparation of methane inventories and thus improve the estimation of methane production in beef and dairy cattle.

## Simple Summary

Enteric methane (CH_4_) production in cattle accounts for a significant portion of global greenhouse gas emissions. Measurement of enteric methane emission is complex, expensive, and large-scale measurement is impractical. Therefore, in the absence of measurements, modeling can be used to predict CH_4_ production and help investigate mitigation options. In this study, we used dietary nutrient composition (g/kg), nutrients (kg/day), energy (MJ/day), and energy and organic matter (OM) digestibility (g/kg) as predictors of CH_4_ production to develop linear and nonlinear statistical models for predicting enteric methane emission from beef and dairy cattle and to evaluate the few available models.

## 1. Introduction

Methane emissions constitute a significant portion of greenhouse gas (GHG) emissions and are crucial in the initiatives aimed at addressing climate change. According to the Paris Agreement, different countries have agreed to limit global temperature rise to 1.5 °C, necessitating a 24–47% reduction in methane emissions from agriculture and establishing a target of net-zero CO_2_ emissions by mid-century [1]. Studies have shown [2,3] that the livestock sector emits about 7.1 billion tonnes of carbon dioxide equivalent of GHG annually, accounting for 14.5% of global anthropogenic GHG emissions. Emissions from cattle are 4.6 gigatonnes, with beef production contributing 2.5 gigatonnes and dairy production 2.1 gigatonnes. About 45% of the GHG emissions from cattle originate from methane produced by enteric fermentation [4].

To better estimate enteric methane emissions from ruminants and explore emission reduction pathways, researchers have developed a variety of dynamic mechanistic and empirical prediction models. These models help to analyze the factors influencing methane production and provide tools for the evaluation of emission reduction strategies. Predictive modeling has been the mainstay of research due to the complexity of directly measuring dietary energy lost as methane. Based on datasets from different research institutions, scientists have developed several statistical models to predict EME and to analyze the effect of dietary composition on rumen fermentation in cattle (e.g., [5,6,7,8,9]). These statistical models can accurately predict methane emission yields from variables such as nutrient intake, food composition, feed levels, and digestibility [10,11,12,13,14,15]. This approach effectively avoids the need for extensive and expensive experiments and provides a scientific basis for the development of methane emission reduction policies [16].

The IPCC (2006) [16] and the Food and Agriculture Organization of the United Nations (FAO) have also developed a model based on the methane emission factor (Ym), which represents the proportion of total energy intake (GE) lost as methane. This model has been widely used in national GHG inventories and global mitigation strategy studies [17]. However, Ym does not directly reflect changes in methane emissions caused by rumen fermentation of different carbohydrates. Therefore, the Ym-based models have limitations in predicting EME and evaluating dietary methane mitigation options [14]. In addition, the weak predictive power of Ym may lead to large errors in the preparation of GHG emission inventories [18,19]. Therefore, further exploration of the relationship between dietary variables and methane production is essential to improve the accuracy of these prediction models [20].

Despite the development of various models to predict EME in beef and dairy cattle, only a few have been able to successfully utilize datasets from different breeds simultaneously. To improve the prediction of enteric methane emissions under different feeding conditions and across breeds, the aims of this study were (1) to develop a prediction model capable of quantifying the effect of diet composition on enteric methane emissions (g/kg DM intake), and (2) to evaluate the predictive ability of this model and other published models using a database of beef and dairy cattle and their combinations.

## 2. Materials and Methods

### 2.1. Construction of Database

A database of beef cattle, dairy cattle, and their combinations was constructed from 149 treatment-averaged data points derived from 34 studies published in journals and conference proceedings, aimed at developing predictive models for intestinal methane production in dairy and beef cattle. These studies included data on animal characteristics, dietary composition, feed intake, nutrient intake and digestibility, and intestinal methane production, measured using respiratory chambers or sulfur hexafluoride tracer technology. The database was subdivided into beef, dairy, and combinations of both (Table 1). The studied animal factors (independent variables) included body weight (BW), dry matter (DM) intake, total energy (GE), metabolizable energy (ME), individual nutrients, chemical composition of diets (crude protein (CP), crude fat (EE), neutral detergent fiber (NDF), acid detergent fiber (ADF), non-fibrous carbohydrates (NFC), organic matter (OM) and GE digestibility, ration proportion and feeding level (FL) to establish regression equations. The chemical composition of the diets was recorded based on the published data from each paper. Some reports were inconsistent or incomplete recorded, necessitating calculations based on the reported data. When chemical composition data were missing, tabulated values [21] were used to calculate the chemical composition of the diets.

When the GE intake (MJ/day) was not reported in publications, it was estimated by the DM intake and GE concentration (MJ/kg DM) calculated from diet chemistry [22]:

$$\mathrm{GE}\;\mathrm{intake}\;(\mathrm{MJ}/\mathrm{day})=\mathrm{DM}\;\mathrm{intake}\;(\mathrm{kg}/\mathrm{day})\times \left[\frac{\left\{23.6\times \mathrm{CP}\;(g/\mathrm{kg})+39.8\times \mathrm{EE}\;(g/\mathrm{kg})+17.3\times \mathrm{NFC}\;(g/\mathrm{kg})+18.9\times \mathrm{NDF}\;(g/\mathrm{kg})\right\}}{1000}\right]$$

When the DE intake (DEI) or ME intake (MEI) was not reported, it was calculated as follows [8]:

DEI(MJ/d)=MEintake(Mj/d)−FE(MJ/d)

$$\mathrm{MEI}(\mathrm{MJ}/d)=\mathrm{DE}\;\mathrm{intake}(\mathrm{MJ}/d)-CH_{4}(\mathrm{MJ}/d)-\mathrm{UE}(\mathrm{MJ}/d)$$

where FE (fecal energy) and UE (urinary energy) represent the energy loss from fecal excretion and urinary excretion, respectively. The feeding level (FL) as a multiplier for maintenance was calculated by dividing the ME intake by the maintenance period requirement [23]:

$$\mathrm{FL}(\mathrm{cattle})=\mathrm{MEI}(\mathrm{MJ}/d)/[0.53\mathrm{MJ}/(\mathrm{kg}/d)]\times [{\left(\mathrm{BW}(\mathrm{kg})/1.08\right)}^{0.67}]/(0.35q+0.503)$$

where q is the metabolic rate (ME:GE).

Only studies that included digestibility data were used in the final analyses. When a study did not report OM digestibility, it was estimated using an equation derived from the data of this study. When either OM or GE digestibility was reported, it was estimated using predictive equations derived from the current dataset as follows.

$$\mathrm{OM}\;\mathrm{digestibility}(g/\mathrm{kg})=40.4(\pm 23.4)+0.966(\pm 0.0334)\times \mathrm{GE}\;\mathrm{digestibility}\left(\mathrm{RMSE}=63.4\right)$$

$$\mathrm{GE}\;\mathrm{digestibility}(g/\mathrm{kg})=9.6(\pm 22.4)+0.84(\pm 0.0313)\times \mathrm{OM}\;\mathrm{digestibility}\left(\mathrm{RMSE}=7.9\right)$$

GE digestibility(g/kg)=16.2(±23.5)+0.765(±0.0337)×DM digestibility(RMSE=32.8)

Since nutrient digestibility varies with feeding levels, digestibility at the maintenance feeding level (OMDm, g/kg) is the most consistent assessment of feed digestibility [8], and the relationship between DM intake/BW and OM digestibility was utilized to adjust the actual OM digestibility to OMDm (1.83/1 g/kg DM intake/BW; [8]) with the following equation.

$$\mathrm{OMDm}(g/\mathrm{kg})=\mathrm{OMdigestibility}(g/\mathrm{kg})+1.83\times \left(\frac{\mathrm{DMinatke}}{\mathrm{BW}}\left(\frac{g}{\mathrm{kg}}\right)\mathrm{at}\;\mathrm{actual}\;\mathrm{level}-\frac{\mathrm{DMinatke}}{\mathrm{BW}}\left(\frac{g}{\mathrm{kg}}\right)\mathrm{at}\;\mathrm{maintenance}\;\mathrm{level}\right)$$

The DM intake/BW (g/kg) at the maintenance level was calculated from prediction equations derived from the dataset of this study.

$$\frac{\mathrm{DM}\;\mathrm{intake}}{\mathrm{BW}}(g/\mathrm{kg})=13.8(\pm 0.8)+2.195(\pm 0.1838)\times \mathrm{FL}(\mathrm{RMSE}=5.7)$$

In the published papers, not all observations of variables in the dataset were useful. So, the number of observations used to develop the predictive equations depended on the available regression variables.

Some records were incomplete or inconsistent and required calculations based on reported data. Conversion units were based on methane production L/d or kcal/day converted to k/d (1 L = 0.714 g methane, 1 L methane = 39.54 KJ energy), g/d converted to kJ/d (1 g methane = 55.65 kJ energy), and energy intake (1 kcal = 4.184 kJ). Missing variables were considered as missing data when a study failed to report a possible outcome, and calculations could not be derived from the reported data.

### 2.2. Statistical Models

#### 2.2.1. Linear Model

The statistical analysis procedures used to predict methane production from this database have been described elsewhere [19]. All statistical calculations were performed using R software (R Core Team, 2023; version 4.4.1, R Foundation for Statistical Computing, Vienna, Austria). The distribution of Cook’s D impact statistics on the data was visually inspected to detect outliers that significantly increased the error variance and to exclude outliers from subsequent analyses. Based on St-Pierre’s (2001) analysis of the data [24], and taking into account the random effects of the study, which represented a random sample of the larger study population, methane prediction equations were developed using the lme4 function with the following model:

$$Y_{ij}=B_{0}+B_{1}X_{ij}+B_{2}X_{ij}^{2}+s_{i}+b_{i}X_{ij}+e_{ij}$$

where *Y_ij_* = expected outcome of the dependent variable *Y* observed at level *j* of the continuous variable *X* in study *i*; *B*0 = intercept (fixed effects) for all studies; *B*_1_ and *B*_2_ = overall linear and quadratic regression coefficients of *Y* on *X* for all studies, respectively (fixed effects); *X_ij_* = value *j* of variable *X* in study *i*. *S_i_* = random effect of study *i*. *b_i_* = random effect of study *i* on the regression coefficient of *Y* on *X* in study *i*, and *e_ij_* = Unexplained residual error.

To address disparities among studies, observed methane production was adjusted based on the number of animals involved in each study. The slopes and intercepts of the studies were included as random effects and the unstructured variance-covariance matrix (type = un) or the variance component of the variance-covariance structure (type = vc) was implemented in the random part of the model (when the random covariance component was not significant and the model could not converge) [24].

Predictors were removed from the model if neither the stochastic covariance nor the stochastic slope and squared terms were significant (*p* > 0.10). Multiple regression equations were further developed using all predictors of methane production using a backward elimination multiple regression procedure, following the algorithm of refs. [19,25]. To avoid the problem of multicollinearity in the fitted model, the variance inflation factor (VIF) for each continuous independent variable tested was considered to be less than 10. The problem of multicollinearity usually arises when the VIF is greater than 10, and if the VIF threshold is set to 1, it may excessively exclude the valid independent variables [26]. The best fitting equations for multiple regression equations that further improve the relationship obtained from simple linear or multiple regression are presented in the paper.

#### 2.2.2. Nonlinear Models

The DM intake was used as a determinant for developing a nonlinear relationship between DM intake and methane emissions to build a nonlinear model if predictions could be further improved. This was performed because there is a high degree of certainty in predicting methane emissions with DM intake as a single independent variable in a linear model, and because the DM intake variable is easy to measure.

The nonlinear model employed relationships exhibiting diminishing returns (single molecule), s-type (Gompertz), and exponential behavior. The equation and exponential model of [27] were slightly modified using R’s linear and nonlinear mixed-effects model/random approximation expectation–maximization algorithm nlme/saemix to parametrize the nonlinear function in the following form:

$$Monomolecular:\;Y=a-\left(a+b\right)\times \exp\left(-c\times x\right)$$

Mitscherlich: Y=a×(1−exp⁡(−c×x))

Gompertz: Y=b×exp ((1−exp (−c×x)×ln (a+2b)/b)−2b)

Exponential: Y=b×exp (c×x)

$$Power:\;Y=b\times x^{c}$$

where Y denotes the predicted methane production, parameters a and b denote the upper asymptote and Y-intercept of the nonlinear model, respectively, and c determines the shape of the response curve in the nonlinear function. Studies that include parameters a, b, and c are considered random in the model [27,28]. The minimum value of the Bayesian information criterion (BIC, a measure of regression fit) and the biological relevance of the estimated parameters were considered to find the best nonlinear model.

#### 2.2.3. Model Evaluation

Three databases were used to evaluate developed and existing models. Thus, inputs from the three databases were used to compare the predictive ability of a range of existing models developed from beef and dairy cattle such as Mills et al. (2003) [5], IPCC (2006) Tier 2 [16], Ellis et al. (2007) [6], Yan et al. (2009) [7], Ramin and Huhtanen (2013) [8], and Patra (2017) [9] (Table 2). These equations were chosen for comparison because they have typically been evaluated in different studies and their input variables are available from this compiled database. The equations developed in this study were compared to the existing equations using the mean squared prediction error (MSPE), the square root of the MSPE (RMSPE) as a percentage of the observed mean [29], and the adjusted coefficient of determination (Adjusted R^2^) [30]. The MSPE values were calculated as:

$$\mathrm{MSPE}=\sum_{i=1}^{n}\;\frac{(O_{i}-P_{i}{)}^{2}}{n}$$

where Oi is the observed value of the ith observation, Pi is the predicted value of the ith observation, and n is the number of observations. The square root of the MSPE (RMSPE) provides an estimate of the overall prediction error, which is expressed as a proportion of the mean of the observations (the MSPE divided by the mean of the observations) to allow comparison of the RMSPE (%) values between equations with different predicted means so that deviations from the observations can be assessed. The MSPE values were decomposed into the average bias or error of the concentrated trend (ECT), the slope bias or error of the regression (ER), and the random bias or error of the disturbance (ED). These three scores are calculated as follows [31]:

$$\mathrm{ECT}={\left(\overset{-}{P}–\overset{-}{O}\right)}^{2}$$

$$\mathrm{ER}={\left(\begin{array}{c} S_{p}-r\times S_{0} \end{array}\right)}^{2}$$

$$\mathrm{ED}=\left(\begin{array}{c} 1–r^{2} \end{array}\right)\times S_{0}^{2}$$

where the entities $\overset{-}{P}$ and $\overset{-}{O}$ are the mean predicted and observed values, respectively; Sp and So are the standard deviations of the predicted and measured values, respectively; and r is the correlation coefficient between the predicted and measured values. ECT values indicate how the mean of the predicted values deviates from the mean of the observed values. Error due to regression measures the deviation of the least squares regression coefficient ($r\times S_{0}/S_{p}$) from 1, the value at which the prediction is perfectly accurate. Larger ER values indicate that the model is underpredicting the variables. Error on perturbations $\overset{-}{P}$ indicates changes in observations that cannot be explained after the mean and regression bias have been removed.

The consistency correlation coefficient (CCC), also known as the reproducibility index, is used to assess the precision and accuracy of the predicted versus measured values for each model [32]. The CCC estimate is expressed as the product of two components. The first component is the correlation coefficient (r) of the measurement accuracy (deviation of observations from the line of best fit). The second component is the bias correction factor (Cb), which indicates how far the regression line deviates from the line of unity (precision). The other estimate (m) measures the positional displacement relative to the scale (the difference between the mean value and the square root of the product of two standard deviations), where negative values indicate overprediction and positive values indicate underprediction of observations by the model.

## 3. Results

### 3.1. Description of Dataset

Table 1 describes the descriptions of feed and animal characteristics included in this database, such as BW, nutrient and energy intake, feed digestibility and methane production. The database was subdivided into beef cattle, dairy cattle, and their combination. The CP and NDF concentrations ranged from 42 to 276 (mean 158) g/kg and 93 to 776 (mean 367) g/kg DM, respectively, suggesting that there was a high degree of variation in the quality of the diets in this database. Mean concentrations of CP and NDF in the rations indicated that the dataset consisted mainly of low to medium-quality rations. The concentrations of EE, although highly variable, had low mean concentrations. Although the proportion of roughage in the rations ranged from 0 to 1000 g/kg, the mean proportion of forage was high (657 g/kg), indicating that the study included mainly forage-based diets. The wide range of digestibility of DM, NDF, CP, and EE in the dataset suggests that digestibility is highly dependent on dietary chemistry. Methane emissions expressed as MJ/day, g/kg DM intake, and the percentage of the total energy intake also varied considerably in the dataset.

### 3.2. Correlation Between Methane Production and Animals and Diets

Consistent with previously published studies [8,9], DMI (Kg/d) and MEI (MJ/d) were the best predictors of CH_4_ production in this study. The joint database study showed that methane production (MJ/d) was positively correlated (*p* = 0.09 to <0.001) with body weight and the intake of all animals (r = 0.39 to 0.91), with DMI and MEI predicting methane production with R^2^ of 0.83 and 0.81, respectively. In the beef database, methane production (MJ/d) was positively correlated (*p* = 0.006 to <0.001) (r = 0.21 to 0.86), where DMI and MEI predicted methane production with R^2^ of 0.68 and 0.63, respectively. In the dairy database, methane production (MJ/d) was positively correlated (*p* = 0.0043 to <0.001) with body weight and intake in all animals (r = 0.59 to 0.9). DMI and MEI predicted methane production with R^2^ of 0.81 and 0.80, respectively (Table 3).

Many previous equations rely on DMI or MEI to predict CH_4_ yield; therefore, we created DMI-based and MEI-based prediction equations in our database for comparison (Table 4). Equations (1b) and (2b) predicted CH_4_ yields for beef cattle using only MEI and DMI, respectively. Equations (1d) and (2d) predicted CH_4_ yields for dairy cattle using only MEI and DMI, respectively. Equations (1c) and (2c) predicted CH_4_ yields from the combined databases using only MEI and DMI, respectively (Table 4), and the results of the RMSPE analyses are shown in Table 5. For all databases, the prediction of CH_4_ yields using DMI resulted in lower RMSPE values and higher R^2^ values than using MEI. Because DMI was reported for all datasets; however, many of the MEI values were extrapolated from other information provided in publications and may contain some errors when compared to the DMI values. Theoretically, the MEI has a stronger relationship with CH_4_ production than the DMI because it takes CH_4_ production into account in its derivation [5].

### 3.3. Prediction Equations for Methane Production

Predictive models for enteric methane emissions were developed for the three databases for beef cattle, dairy cattle, and a combination of the two using BW, DM intake, diet composition for nutrients (NDF and ADF), energy (GE and ME) and nutrients (CP, EE, NDF, ADF and NFC) as predictors (Table 4). When methane production was predicted using a single predictor variable in each database, intake of DM ((1c) R^2^ = 0.79, (1b) R^2^ = 0.69, and (2d) R^2^ = 0.84) was a high predictor of methane production, but intake of ME ((2c) R^2^ = 0.65, (2b) R^2^ = 0.66, and (3d) R^2^ = 0.58) and NDF ((3c) R^2^ = 0.78, (3b) R^2^ = 0.73, and (4d) R^2^ = 0.74) intake predicted low or moderate methane production. Among nutrients (CP, EE, NDF, ADF, and NFC), ADF and NFC concentrations predicted methane production, but at low predictive values. CP, NFC and EE concentrations in this database did not predict methane emissions.

The multiple regression model with DM and NDF, ADF intake, ME and NDF, and ADF intake as the two explanatory variables in the model, slightly improved methane yield prediction over the model with DM intake as the single predictor variable, such that Equation (4b) added the variable NDFI to the univariate DMI of (1b) (both R-squared 0.83 and RMSEs of 1.57 and 2.13), does improve the model predictions to some extent, but the magnitude of this improvement is limited. Multiple regression equations that included DM intake, NDF intake, and ADF intake slightly improved predictions ((8c) R^2^ = 0.85, (8b) R^2^ = 0.83, and (9d) R^2^ = 0.88), and none of the other independent variables or interactions significantly improved methane emission predictions.

Nonlinear models of DM and ME intake were used to predict methane emissions because ME intake was the strongest predictor as a single factor in methane prediction and dry matter intake was easy to measure. However, the Gompertz model did not significantly improve the prediction of methane emissions compared to a linear model using DM intake ((1c) R^2^ = 0.79, (1b) R^2^ = 0.69, and (2d) R^2^ = 0.84) as a single predictor. Exponential growth ((10c) R^2^ = 0.81, (9b) R^2^ = 0.73, and (12d) R^2^ = 0.86) and power ((11c) R^2^ = 0.84, (10b) R^2^ = 0.67, and (14d) R^2^ = 0.88) models further improved the R^2^ for methane prediction compared to the linear model with lower RMSE values.

**Table 3:** Correlations between dietary variables and CH_4_ production (MJ/d) for the beef, dairy, and combined (beef and dairy) databases.

Variable	r	*p*-Value	R^2^	r	*p*-Value	R^2^
Beef						
Body weight (kg)	0.46	<0.001	0.21			
DM intake, kg/d	0.83	<0.001	0.68			
GE intake, MJ/d	0.79	<0.001	0.62			
ME intake, MJ/d	0.8	<0.001	0.63			
	Intake (kg/day)			Nutrient concentration (g/kg DM)		
Crude protein	0.62	<0.001	0.39	0.02	0.821	0.04
Ether extract	0.3	0.006	0.29	−0.1	0.363	0.01
Neutral detergent fiber	0.86	<0.001	0.75	0.11	0.329	0.11
Acid detergent fiber	0.21	0.06	0.44	−0.09	0.404	0.07
Non-fibrous carbohydrate	0.53	<0.001	0.28	−0.1	0.375	0.016
Dairy						
Body weight (kg)	0.77	<0.001	0.56			
DM intake, kg/d	0.9	<0.001	0.81			
GE intake, MJ/d	0.75	<0.001	0.6			
ME intake, MJ/d	0.89	<0.001	0.8			
	Intake (kg/day)			Nutrient concentration (g/kg DM)		
Crude protein	0.86	<0.001	0.74	0.4	0.001	0.16
Ether extract	0.63	<0.001	0.4	−0.06	0.63	0.07
Neutral detergent fiber	0.86	<0.001	0.73	−0.38	0.002	0.14
Acid detergent fiber	0.59	0.004	0.34	−0.32	0.01	0.1
Non-fibrous carbohydrate	0.76	<0.001	0.57	0.11	0.403	0.09
Combined						
Body weight (kg)	0.7	<0.001	0.49			
DM intake, kg/d	0.91	<0.001	0.83			
GE intake, MJ/d	0.74	<0.001	0.55			
ME intake, MJ/d	0.87	<0.001	0.81			
	Intake(kg/day)			Nutrient concentration (g/kg DM)		
Crude protein	0.84	<0.001	0.7	0.19	0.019	0.04
Ether extract	0.39	0.09	0.35	−0.21	0.009	0.05
Neutral detergent fiber	0.9	<0.001	0.8	0.03	0.713	0.194
Acid detergent fiber	0.53	<0.001	0.48	−0.23	0.004	0.05
Non-fibrous carbohydrate	0.73	<0.001	0.53	−0.09	0.02	0.3

DM, dry matter; GE, gross energy; ME, metabolizable energy; CP, crude protein.

### 3.4. Comparison of Models

The established and existing methane prediction equations from the beef, dairy, and combined databases are shown in Table 4, and the results of the RMSPE and CCC analyses are shown in Table 5. For the beef database, the univariate equations (Equations (1b)–(3b)) showed that DMI and NDFI were the best predictors of CH_4_ production in terms of RMSPE (RMSPE% = 30.07 and 27.75, respectively), with high precision (CCC values = 0.8 and 0.85, respectively) and accuracy (Cb values = 0.82 and 0.85, respectively). In the equations containing two variables (Equations (4b)–(7b)), including DM intake and NDF intake, ME intake and NDF intake, had the lowest RMSPE values (RMSPE% = 22.19 and 22.16, respectively), high precision (CCC values = 0.91 and 0.85, respectively) and accuracy (Cb values = 0.89 and 0.89). The equation containing the three variables DM intake, NDF intake, and ADF intake (Equation (8b)) had a lower RMSPE value (RMSPE% = 21.99), higher precision (CCC value = 0.91) and accuracy (Cb value = 0. 9). Among the nonlinear models, the exponential model was slightly higher than the univariate prediction linear and nonlinear models in terms of RMSPE (29.38%, random error 75.99%, regression bias 24.01%), precision (CCC = 0.79) and accuracy (Cb value = 0. 72). Since the number of treatments varied between variables depending on the available data, the precision and accuracy between models may cause some bias in model comparisons.

**Table 4:** List of statistical models developed to predict intestinal methane production (MJ/day) based on three databases.

Database	Equation No.	Equation: Methane (MJ/day)
Beef	Equation (1b)	= 0.9188 (± 0.5849) + 0.8196 (± 0.069) × DMI (kg/day); RMSE = 2.13,R^2^ = 0.83
	Equation (2b)	= 2.4904 (± 0.5029) + 0.0657 (± 0.0059) × MEI (MJ/day); RMSE = 2.24, R^2^ = 0.76
	Equation (3b)	= 2.5492 (± 0.4222) + 1.8401 (± 0.1384) × NDFI (kg/day); RMSE = 1.96, R^2^ = 0.73
	Equation (4b)	= 0.9462 (± 0.4351) + 0.4368 (± 0.0739) × DMI (kg/day) + 1.1567 (± 0.1606) × NDFI (kg/day); RMSE = 1.57, R^2^ = 0.83
	Equation (5b)	= 0.019 (± 0.971) + 0.8058 (± 0.0698) × DMI (kg/day) + 0.649 (± 0.5599) × ADFI (kg/day); RMSE = 2.11, R^2^ = 0.7
	Equation (6b)	= −0.8848 (± 0.9411) + 0.0669 (± 0.0053) × MEI + 2.1255 (± 0.5205) × ADFI (kg/day); RMSE = 1.98, R^2^ = 0.73
	Equation (7b)	= 1.6836 (± 0.3698) + 0.0342 (± 0.0058) × MEI + 1.2202 (± 0.1526) × NDFI (kg/day); RMSE = 1.57, R^2^ = 0.83
	Equation (8b)	= 1.6063 (± 0.757) + 0.4256 (± 0.0745) × DMI (kg/day) + 1.2213 (± 0.1715) × NDFI (kg/day) + −0.475 (± 0.446) × ADFI (kg/day); RMSE = 1.55, R^2^ = 0.83
	Exponential 1	= −1.264 (± 0.1663) × exp{0.3094 (± 0.0243)} × DMI (kg/day); RMSE = 1.21, R^2^ = 0.73
	Exponential 2	= −3.002 (± 0.8369) × exp{0.0172 (± 0.0076)} × MEI (MJ/day); RMSE = 2.33, R^2^ = 0.70
	Power	= 1.039 (± 0.1526) × DMI (kg/day) ^ 0.9474 (± 0.0601); RMSE = 2.28, R^2^ = 0.67
Dairy	Equation (1d)	= −6.9931 (± 3.2072) + 0.0448 (± 0.0059) × BW (kg); RMSE = 4.58, R^2^ = 0.55
	Equation (2d)	= 0.7567 (± 1.1008) + 1.0917 (± 0.0703) × DMI (kg/day); RMSE = 2.73, R^2^ = 0.84
	Equation (3d)	= 5.5616 (± 1.5382) + 0.0789 (± 0.0099) × MEI(MJ/day); RMSE = 4.43, R^2^ = 0.79
	Equation (4d)	= 1.6799 (± 1.3882) + 2.7698 (± 0.2388) × NDFI (kg/day); RMSE = 3.45, R^2^ = 0.74
	Equation (5d)	= 0.3989 (± 1.1073) + 0.8685 (± 0.1585) × DMI (kg/day) + 0.6675 (± 0.4264) × NDFI (kg/day); RMSE = 2.66, R^2^ = 0.85
	Equation (6d)	= 0.8642 (± 1.2845) + 1.1024 (± 0.0953) × DMI (kg/day) + −0.1051 (± 0.6271) × ADFI (kg/day); RMSE = 2.73, R^2^ = 0.84
	Equation (7d)	= 2.9962 (± 1.9512) + 0.0646 (± 0.0119) × MEI (MJ/day) + 1.8279 (± 0.8999) × ADFI (kg/day); RMSE = 4.24, R^2^ = 0.62
	Equation (8d)	= 1.7105 (± 1.4001) + 0.0084 (± 0.0152) × MEI (MJ/day) + 2.5447 (± 0.4704) × NDFI (kg/day); RMSE = 3.43, R^2^ = 0.75
	Equation (9d)	= 0.5246 (± 1.2833) + 0.8805 (± 0.171) × DMI (kg/day) + 0.6691 (± 0.4312) × NDFI (kg/day) + −0.1237 (± 0.6174) × ADFI; RMSE = 2.65, R^2^ = 0.88
	Equation (10d)	= −3.0617 (± 2.218) + 0.0147 (± 0.0144) × MEI (MJ/day) + 2.4028 (± 0.444) × NDFI (kg/day) + 0.007 (± 0.0026) × rf; RMSE = 3.18, R^2^ = 0.78
	Gompertz	= 1.396 (± 2.41) × exp([1 − exp{−0.1002 (± 0.0292)} × DMI (kg/day)] × ln{39.31 (± 5.48) + 2 × 1.396 (± 2.41)/1.396 (± 2.41)}) – 2 × 1.396 (± 2.41); RMSE = 3.34, R^2^ = 0.8
	Exponential 1	= –4.076 (± 0.3476) × exp{0.0765 (± 0.0071)} × DMI (kg/day); RMSE = 1.27, R^2^ = 0.86
	Exponential 2	= −4.84 (± 0.3918) × exp{0.0055(± 6 × 10^−4^)} × MEI (MJ/day); RMSE = 1.5, R^2^ = 0.69
	Power	= 0.887 (± 0.177) × DMI (kg/day) ^ 1.085 (± 0.0694); RMSE = 2.62, R^2^ = 0.88
Combined	Equation (1c)	= 0.0529 (± 0.6167) + 1.0469 (± 0.0538) × DMI (kg/day); RMSE = 3.04, R^2^ = 0.83
	Equation (2c)	= 3.88 (± 1.313) + 0.071 (± 0.011) × MEI (MJ/day); RMSE = 1.36, R^2^ = 0.81
	Equation (3c)	= 0.9454 (± 0.5986) + 2.6638 (± 0.1424) × NDFI (kg/day); RMSE = 1.43, R^2^ = 0.78
	Equation (4c)	= −0.3821 (± 0.5224) + 0.5932 (± 0.0835) × DMI (kg/day) + 1.3849 (± 0.2141) × NDFI (kg/day); RMSE = 0.54, R^2^ = 0.85
	Equation (5c)	= −0.6042 (± 0.8538) + 1.0088 (± 0.0638) × DMI (kg/day) + 0.5422 (± 0.4879) × ADFI (kg/day); RMSE = 1.45, R^2^ = 0.79
	Equation (6c)	= −0.2924 (± 1.1841) + 0.0702 (± 0.0077) × MEI (MJ/day) + 2.3215 (± 0.6256) × ADFI (kg/day); RMSE = 1.52, R^2^ = 0.61
	Equation (7c)	= 0.7085 (± 0.5998) + 0.0167 (± 0.0081) × MEI (MJ/day) + 2.3096 (± 0.2211) × NDFI (kg/day); RMSE = 1.56, R^2^ = 0.79
	Equation (8c)	= −0.3496 (± 0.723) + 0.5941 (± 0.0851) × DMI (kg/day) + 1.388 (± 0.2203) × NDFI (kg/day) + −0.0276 (± 0.4223) × ADFI (kg/day); RMSE = 1.47, R^2^ = 0.85
	Gompertz	= 0.956 (± 0.732) × exp([1 − exp{−0.1058 (± 0.0144)} × DMI (kg/day)] × ln{36.23 (± 2.59) + 2 × 0.956 (± 0.732)/0.956 (± 0.732)}) − 2× 0.956 (± 0.732); RMSE = 3.34, R^2^ = 0.74
	Exponential	= −3.667 (± 0.2125) × exp{0.0855 (± 0.0051)} × DMI (kg/day); RMSE = 1.62, R^2^ = 0.81
	Power 1	= 0.782 (± 0.0938) × DMI (kg/day) ^ 1.1087 (± 0.0435); RMSE = 2.63, R^2^ = 0.84
	Power 2	= 0.205 (± 0.0508) × MEI (MJ/day) ^ 0.8528 (± 0.049); RMSE = 3.58, R^2^ = 0.7

For the dairy database, tests of the univariate equations (Equations (1d)–(4d)) showed that DMI and NDFI were the best predictors of CH_4_ production in terms of RMSPE (RMSPE% = 16.43 and 20.74, respectively), with a high degree of precision (CCC values = 0.91 and 0.86, respectively) and accuracy (Cb values = 0.89 and 0.82). In the equations containing two variables (Equations (5d)–(8d)), the equations including DM intake and NDF and ADF intake, had the lowest RMSPE values (RMSPE% = 15.99 and 16.42, respectively), high precision (CCC values = 0.92 and 0.91, respectively) and accuracy (Cb values = 0.82 and 0.75, respectively). The equation containing three variables of ME intake, NDF intake, and roughage (Equation (10d)) had lower RMSPE values (RMSPE% = 19.14 and random error of 78.65). Among the nonlinear models, the power model was slightly higher than the linear and nonlinear models for univariate prediction in terms of RMSPE (18.23%, random error 88.57%, and regression bias 12.43%), precision (CCC = 0.89), and accuracy (Cb value = 0.72). Increasing the complexity of the beef database equations decreased the RMSPE values, but increasing the complexity of the dairy database did not significantly improve the predicted values.

When the beef and dairy databases were combined, tests of the univariate equations (Equations (1c)–(3c)) showed that DMI was the best predictor of CH_4_ production in terms of RMSPE (RMSPE% = 29.2, random error 79.41%, regression bias 20.59%), with a high degree of precision (CCC value = 0.84) and accuracy (Cb value = 0.92). In the equations with two variables (Equations (4c)–(7c)), including DM intake and NDF intake had the lowest RMSPE value (RMSPE% = 24.41, stochastic error 85.62%, and regression bias 14.38%), high precision (CCC value = 0.89), and accuracy (Cb value = 0.92). Among the nonlinear models, the exponential model was slightly higher than the univariate predicted linear and nonlinear models in terms of RMSPE (25.72%, random error 83.31%, regression deviation 16.69%), precision (CCC = 0.89) and accuracy (Cb value = 0.91). No additional advantage in terms of RMSPE was gained by increasing the complexity of the combined database equations, possibly due to the legacy effects of the dairy database. Some of the simpler equations had lower RMSPE values than the more complex ones; therefore, they are not presented here.

**Table 5:** Mean square prediction error analysis using developed and extant methane prediction equations.

Dataset	Equation No.	RMSPE%	ECT%	ER%	ED%	r	CCC	Cb	μ
Beef	Equation (1b)	30.07	0.04	34.01	69.16	0.81	0.8	0.82	−0.01
	Equation (2b)	31.58	0.07	30.84	65.99	0.83	0.82	0.83	−0.05
	Equation (3b)	27.75	0.71	26.27	73.73	0.86	0.85	0.85	−0.11
	Equation (4b)	22.19	0.16	16.79	83.21	0.91	0.91	0.89	0.05
	Equation (5b)	29.75	0.03	30.19	69.81	0.84	0.86	0.83	0.05
	Equation (6b)	28.03	0.15	26.8	73.2	0.86	0.82	0.85	−0.03
	Equation (7b)	22.16	0.06	16.75	83.25	0.91	0.85	0.89	−0.1
	Equation (8b)	21.99	0.2	16.49	83.51	0.89	0.91	0.9	0.01
	Exponential 1	29.38	0.14	24.01	75.99	0.87	0.79	0.72	−0.05
	Exponential 2	30.1	0.22	24.99	75.01	0.85	0.77	0.87	−0.38
	Power	32.28	0.31	29.24	70.76	0.82	0.88	0.82	−0.19
	Mills et al. (2003) [5] Equation (1)	87.1	76.3	27.07	72.93	0.83	0.39	0.85	−0.98
	Mills et al. (2003) [5] Equation (3)	61.15	80.8	54.14	45.86	0.8	0.61	0.93	−0.79
	IPCC (2006) [16]	48.95	33.7	66.94	33.06	0.79	0.72	0.98	−0.53
	Ellis et al. (2007) [6]	39.85	54.3	53.16	46.84	0.83	0.71	0.98	−0.9
	Yan et al. (2009) [7]	72.74	63.7	55.2	44.8	0.83	0.59	0.91	−0.71
	Ramin and Huhtanen (2013) [8]	55.67	69.6	50.1	49.9	0.78	0.61	0.94	−0.62
	Patra (2015) [9]	34.04	75.1	65.22	34.78	0.8	0.78	0.96	0.57
Dairy	Equation (1d)	27.53	0.11	44.17	55.83	0.75	0.76	0.81	0.03
	Equation (2d)	16.43	0.05	15.72	84.28	0.92	0.91	0.89	0.01
	Equation (3d)	26.67	0.21	41.43	58.57	0.77	0.74	0.86	−0.01
	Equation (4d)	20.74	0.13	25.06	74.94	0.87	0.86	0.82	−0.15
	Equation (5d)	15.99	0.34	14.89	85.11	0.92	0.92	0.82	−0.06
	Equation (6d)	16.42	0.22	15.71	84.29	0.92	0.91	0.75	0.01
	Equation (7d)	25.5	0.08	37.88	62.12	0.79	0.77	0.88	0.04
	Equation (8d)	20.66	0.19	24.88	75.12	0.87	0.86	0.91	−0.11
	Equation (9d)	16.98	0.26	14.88	85.12	0.92	0.92	0.93	−0.07
	Equation (10d)	19.14	0.09	21.35	78.65	0.89	0.88	0.67	0.02
	Gompertz	23.2	0.19	13.16	86.84	0.88	0.86	0.66	−0.02
	Exponential 1	17.23	0.11	11.91	82.26	0.89	0.9	0.7	−0.8
	Exponential 2	32.11	0.13	32.06	87.94	0.81	0.79	0.65	−0.71
	Power	18.23	0.19	12.43	88.57	0.94	0.89	0.72	0.32
	Mills et al. (2003) [5] Equation (1)	27.75	79.3	59.65	40.35	0.9	0.78	0.92	−0.33
	Mills et al. (2003) [5] Equation (3)	23.38	80.2	74.02	25.98	0.89	0.85	0.89	−0.39
	IPCC (2006) [16]	22.32	35.9	81.71	18.29	0.89	0.88	0.94	−0.29
	Ellis et al. (2007) [6]	27.48	62.5	49.1	50.9	0.9	0.74	0.95	−0.62
	Yan et al. (2009) [7]	35.4	71.1	67.77	32.23	0.9	0.77	0.97	−0.46
	Ramin and Huhtanen (2013) [8]	22.03	76	67.9	32.1	0.88	0.84	0.88	−0.33
	Patra (2015) [9]	31.82	61.9	43.14	56.86	0.89	0.68	0.91	0.72
Combined	Equation (1c)	29.2	0.02	20.59	79.41	0.89	0.84	0.92	−0.01
	Equation (2c)	42.9	0.42	44.51	55.49	0.74	0.79	0.89	−0.03
	Equation (3c)	30.1	0.05	21.87	78.13	0.88	0.82	0.91	−0.02
	Equation (4c)	24.41	0.17	14.38	85.62	0.93	0.89	0.92	−0.02
	Equation (5c)	29.02	0.09	20.33	79.67	0.89	0.84	0.91	−0.01
	Equation (6c)	40.19	0.29	38.98	61.02	0.78	0.76	0.85	−0.03
	Equation (7c)	29.46	0.42	20.95	79.05	0.89	0.83	0.92	−0.02
	Equation (8c)	24.4	3.5	14.38	85.62	0.93	0.89	0.92	−0.04
	Gompertz	34.34	0.17	15.51	82.48	0.85	0.85	0.88	−0.03
	Exponential	25.72	0.23	16.69	83.31	0.87	0.89	0.91	0.01
	Power 1	26.98	0.15	14.88	85.12	0.82	0.88	0.91	−0.44
	Power 2	36.74	0.19	27.72	72.28	0.84	0.77	0.78	0.32
	Mills et al. (2003) [5] Equation (1)	50.17	82.4	49.37	50.63	0.91	0.7	0.94	−0.76
	Mills et al. (2003) [5] Equation (3)	37.34	76.2	72.11	27.89	0.9	0.83	0.98	−0.91
	IPCC (2006) [16]	32.09	28.1	81.64	18.36	0.9	0.88	0.99	−0.45
	Ellis et al. (2007) [6]	32.75	66.5	59.41	40.59	0.91	0.81	0.97	−0.89
	Yan et al. (2009) [7]	49.13	69.3	70.18	29.82	0.91	0.79	0.95	−1.21
	Ramin and Huhtanen (2013) [8]	34.43	56.7	67.54	32.46	0.89	0.83	0.99	−0.9
	Patra (2015) [9]	34.74	57.4	59.53	40.47	0.9	0.8	0.99	0.49

Overall, Equation (8b) had the lowest RMSPE values for the beef database, Equation (5d) had the lowest RMSPE values for the dairy database, and Equation (4c) had the lowest RMSPE values for the combination database. The lower RMSPE values obtained for the more complex equations in the beef database may be due to the wider range of diets in the beef database and, therefore, greater variability. It may also be due to the relatively weaker relationship between CH_4_ production and DMI in the beef database compared to the dairy database. In this case, the inclusion of other variables in the model improved its predictive power. Since the number of treatments varies between variables depending on the available data, the precision and accuracy between models may cause some bias in model comparisons.

**Figure 1:**
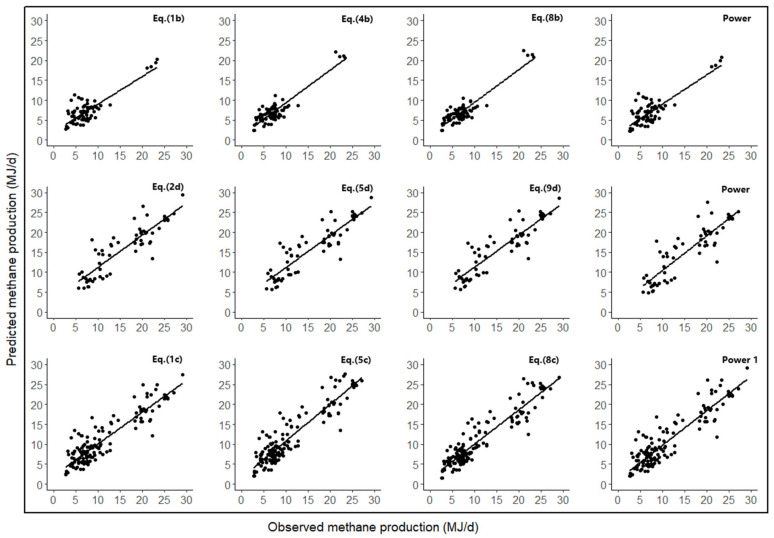
Plots of predicted versus observed (MJ/day) methane production for the new equation.

**Figure 2:**
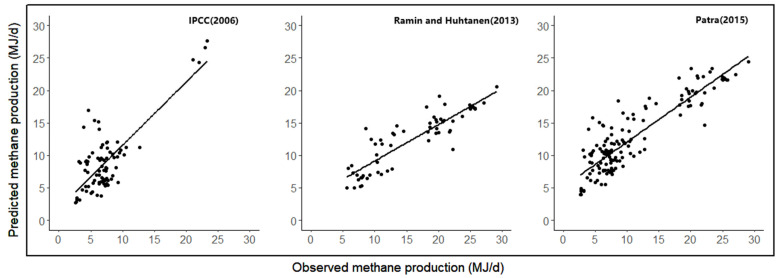
Plots of predicted versus observed (MJ/day) methane production for existing equations [8,9,16].

Figure 1 shows a plot of predicted methane production versus observed methane production for some of the formulas. It shows that the observed and predicted methane yields are well related to low RMSPE, high precision, and predictability. Methane emissions are overestimated by all existing models in the three databases except for the model described by Patra (2015) [9], which exhibits high negative μ values (0.29 to 1.21). Existing models typically have large RMSPE values and large mean biases. Among the existing models, the IPCC (2006) [16] model had a low mean bias, and the Mills et al. (2003) [5] linear model had the largest mean bias (80% of RMSPE). The IPCC (2006) [16] model also had better precision (CCC) and accuracy (Cb) among the existing models. A plot of predicted methane production versus observed methane production for the existing models is shown in Figure 2.

## 4. Discussion

Mean methane yields in the three datasets used for model development were 1.03, 1.12, and 1.07 MJ/kg DM intake, respectively, which were lower than the values reported by previous studies for dairy and beef cattle (ranging from 1.12 to 1.49 MJ/kg DM intake) [6,7,8]. The mean concentrations of CP and NDF in the rations were 159 and 368 g/kg DM, respectively, suggesting that the lower methane production (MJ/kg DM intake) in this study may be due to the inclusion of low to medium rations in the three datasets, and hence the lower digestibility (mean digestibility 69%).

**Figure 3:**
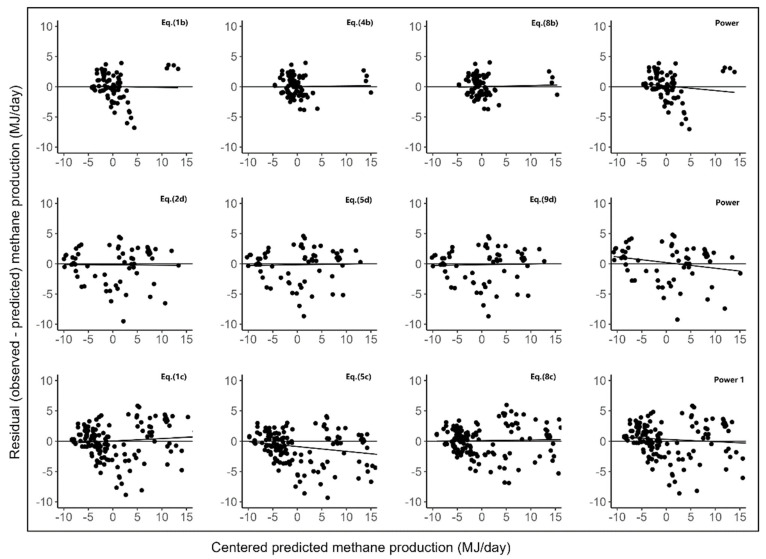
Plots of the observed minus predicted methane production (residual) vs. predicted methane production. The independent variable (predicted methane production) was centered around the mean predicted value before the residuals were regressed on the predicted values.

Various models developed in this study indicated that nutrient intake was a stronger determinant of CH_4_ production than nutrient composition. The DM intake or energy intake as a single predictor was more strongly associated with CH_4_ production than other predictors. Many studies have also reported several predictive equations that use intake (DM or energy) as the main predictor of methane yield in dairy and beef cattle [5,7,9,33] with high accuracy. In this study, the R^2^ values for the relationship between methane and DM or ME intake were all between 0.76 and 0.83, which was moderately high. In a previous feeding study on beef cattle in North America [6], predictive equations using DM intake or ME intake as the main predictor of CH_4_ emissions had low R^2^ values (0.44 or 0.36), whereas in another feeding study in the UK, the R^2^ value was moderate (0.68 for DM intake) [7]. The lower variability in diet chemistry (particularly NDF and ADF content and DM intake) in the present dataset compared to the datasets of Yan et al. (2009) and Ellis et al. (2007) [6,7] may have resulted in a better relationship between CH_4_ emissions and nutrient intake. It is expected that ME intake may be a better determinant of CH_4_ production than DM intake because the former takes CH_4_ production into account in its derivation [5]. However, ME intake was less accurate in predicting CH_4_ production compared to DM intake. A possible reason for this may be because the dataset of this study incorporates calculated ME values from several studies rather than direct observations from the studies, which results in errors in the ME values. Nevertheless, Eills et al. (2007) [6] also showed that the predictability of CH_4_ using ME intake was lower than DM intake in the dairy (R^2^ = 0.64 vs. 0.53) and beef cattle (R^2^ = 0.44 vs. 0.36) datasets. The squared term of the dependent variable was not significant (*p* > 0.05) in predicting CH_4_ emissions in this study. However, Ramin and Huhtanen (2013) [8] demonstrated that a quadratic model utilizing DM intake as a single predictor variable showed improved goodness of fit compared to a linear model, but did not perform better than the newly developed equation. A plot of observed methane production minus predicted methane production (residual) versus predicted buffalo methane production is shown in Figure 3.

The chemical composition of the diet and the animal’s BW were used to establish some predictive equations, which may be useful in cases where intake data are not available. However, animal BW, NDF, and ADF concentrations as single determinants were less accurate in predicting methane production. Dietary fat was negatively correlated with methane production, and predictive models for methane production used fat as a predictor when databases included studies of dietary ether extract supplements [34,35]. However, fat was not associated with methane production in this study, which may be due to the presence of low concentrations of EE in the feed. Ellis et al. (2007) [6] reported that multiple regression equations containing DM intake and fat intake improved the predictive model in the beef cattle database, but not in the dairy cattle database.

Multiple regression equations containing DM and NDF intake, DM and ADF intake, or DM, NDF, and ADF intake improved the prediction of methane emissions to some extent (R^2^ = 0.77 to 0.88). The multivariate regression model developed by Ellis et al. (2007) [6] had the highest R^2^ values, which included ME intake, ADF intake, and lignin intake as determinants, with R^2^ = 0.85 for the beef cattle dataset, 0.65 for the dairy cattle dataset, and 0.71 for the combined beef and dairy cattle dataset. In the current database, lignin was not assessed because most studies did not report dietary lignin concentrations. In the present study, multiple regression equations with DM intake, NDF intake, and ADF intake as determinants had R^2^ = 0.83 for the beef cattle dataset, R^2^ = 0.88 for the dairy cattle dataset, and R^2^ = 0.85 for the combined beef and dairy cattle dataset.

The reason for the low correlation of DMI and MEI with CH_4_ in beef databases is not clear. Ellis et al. (2009) [6] showed that beef cattle feedlot DMI was highly correlated with CH_4_ production [12]. However, Basarab et al. (2005) demonstrated that different categories of beef animals, classified by animal type, physiological status, sex, body weight, growth rate, activity level, and age, produced different amounts of CH_4_ [36]. Merging all animals in these categories may lead to the fragmentation of the DMI and CH_4_ relationship in the beef database. A less physiological state and dietary diversity in the dairy database could explain the higher correlation of CH_4_ with DMI and MEI.

The CH_4_ production from the rumen depends on dietary factors, rumen function, and fermentation kinetics [37]. Therefore, methane production may not follow a linear trend as influenced by these factors. This paper also evaluated nonlinear regression models in predicting CH_4_ emissions using DM and ME intake. Among the nonlinear models, the exponential and power models were found to be more precise and accurate compared to simple linear models. Mills et al. (2003) [5] pointed out small differences in RMSPE percentages between linear and nonlinear models. However, nonlinear models may be more appropriate and reliable for predicting CH_4_ emissions over a large range of intake and dietary variables (Mills et al., 2003) [5]. Particularly, they may be more appropriate when the range of data is outside the scope of the database as reported in the present study. Nonetheless, CH_4_ production predictions should be made with caution when the dependent variable is outside the range of this database, as few models have biologically irrelevant intercept values.

The current datasets were used to validate a range of existing equations for predicting CH_4_ production in dairy and beef cattle. The existing equations were developed using CH_4_ yield database on beef cattle (Yan et al., 2009) [7], dairy cattle (Mills et al., 2003) [5], dairy and beef cattle (Ellis et al., 2007) [6], and the combination of cattle and sheep (Ramin and Huhtanen, 2013 [8]). The IPCC (2006) [16] Tier 2 model was selected due to its widespread adoption by countries for the development of CH_4_ emission inventories. The newly developed models performed better than the existing models, because except for the equations for NDF and ADF concentration predictions, the equations had the lowest RMSPE values compared to the existing models evaluated with this database. Although these new equations performed better than the existing models, it would be desirable to also challenge the existing equations on an external database. Inaccuracies and imprecision were higher in the existing North American and European cattle models compared to the new equations developed in this study. These variations can be attributed to differences in animal type, geography, and diet. The diet quality in this study was low to medium, whereas the diet quality in North American and European countries was medium to high. Furthermore, there were also differences in rumen fermentation, nutrient utilization, and microbial populations between cattle and buffaloes [38,39].

The comparison of RMSPE, MSPE scale, CCC, position (v), and scale offset (μ) values of the predictive equations assessed in this study reveals that several linear and nonlinear equations exhibit similar predictive performance in the cattle study. The average RMSPE(%) values for the beef database were 27.7 and 57.1 for the new and existing equations, respectively. For the dairy database, the average RMSPE(%) values were 21.1 and 27.2 for the new and existing equations, respectively. For the integrated database, the average RMSPE(%) values for the new and existing equations were 31.1 and 38.7, respectively, and the overall accuracy of the models decreased, probably due to the physiological differences in the different breeds and the feed intake characteristics that affected the model accuracy. For all three databases, numerical reductions in RMPSE were obtained using the newly developed equations. Therefore, the new simple fitting model developed in this study was able to estimate methane emissions more accurately than existing models that tend to underestimate methane emissions. For the applicability of the newly developed model in this study under conditions such as high-quality feeds, other regions, or different feed compositions and different species, we need to further validate it using external databases to enhance the broad applicability of the model.

## 5. Conclusions

In this study, linear models using DM intake or ME intake as a single predictor were developed based on a database of beef and dairy cattle and their combinations, which improved the prediction of CH_4_ production. In addition, multiple regression equations using DM, NDF, and ADF intakes further improved the model fit and showed higher precision and accuracy compared to the linear model. Among the nonlinear models, the exponential and power models outperformed the traditional linear models.

The existing CH_4_ emission prediction models have a tendency to overestimate, and this problem was reassessed and improved in this study. Most of the equations have low precision and accuracy in CH_4_ emission prediction, except for the IPCC (2006) model [16]. IPCC (2006) [16] proposed a methane emission factor (Ym)-based approach to estimate enteric CH_4_ emissions from ruminants. However, Ym cannot accurately reflect the effects of different carbohydrate types and feeding levels on rumen fermentation. Therefore, Ym-based models have limited application in predicting methane emissions and evaluating methane mitigation options [14]. The equations proposed in this study can better estimate country-specific Ym and CH_4_ emission factors from feed intake and diet composition characteristics. This allows for more accurate methane emission inventories for beef and dairy cattle and avoids over-reliance on the IPCC (2006) [16] default methane emission factors. In addition, this study further reveals the influence of diet composition on CH_4_ production in beef and dairy cattle, which promotes CH_4_ emission reduction in related fields. Nevertheless, validation of these new models in external databases is necessary to evaluate their fit and predictive accuracy across different conditions. This will ensure that the accuracy of these equations is maintained in a wider range of applications.

## Figures and Tables

**Table 1:** Summary of the beef, dairy, and combined databases.

Variable	Database											
	Beef				Dairy				Combined			
	Mean	Min	Max	SD	Mean	Min	Max	SD	Mean	Min	Max	SD
Body weight (kg)	404.58	163	616	120.2	515.9	263	734	118.37	453.15	163	734	131.55
Intake (kg/day or MJ/day)												
DM	7.45	2.29	23.7	4.07	13.92	4.8	26.2	5.79	10.28	2.29	26.2	5.86
GE	134.29	41.5	426	75.77	259.31	87.4	491.6	114.59	188.83	41.5	491.6	113.17
DE	93.91	30.4	328	55.36	176.55	63.8	341.3	84.00	128.55	30.4	341.3	80.00
ME	70.75	22.2	285	49.73	133.91	25.1	262.9	73.93	93.72	22.2	285	66.97
Feeding level ^a^	2.78	0.47	10.47	1.94	4.90	0.49	12	3.34	3.56	0.47	12	2.74
Chemical composition (g/kg)												
OM	922	831	963	23.02	918.66	815	965	40.87	920.90	815	965	32.11
CP	153.78	42	276	52.07	165.29	96.3	210	24.76	158.80	42	276	42.76
EE	55.18	8	318	43.99	36.42	21	62.6	11.15	47.00	8	318	35.10
NDF	352.65	93	776	169.13	386.43	271	599	70.16	367.39	93	776	136.22
ADF	273.43	73	491	106.36	219.85	131	347	53.60	250.47	73	491	91.65
NFC	361	93.4	644	140.04	328.95	150	464	77.22	347.02	93.4	644	117.94
Roughage proportion (g/kg)	682.51	100	1000	250	624.68	50	1000	242.59	657.28	50	1000	248.46
Digestibility (g/kg)												
DM	673.32	493	837	81.57	713.26	543	819	58.78	690.74	493	837	75.17
OM	698.16	503	837	76.52	721.91	595	826	62.37	708.81	503	837	71.51
GE	692.41	496	866	74.86	708.38	566	804	62.19	698.32	496	866	70.86
OMDm	713.52	500	861	78.4	772.18	603	859	56.41	734.34	500	861	76.70
CP	650.06	401	841	95.41	686.59	407	839	82.99	670.05	401	841	90.67
NDF	539.31	236	740	116.66	581.02	403	718	96.44	556.35	236	740	110.77
ADF	433.92	244	658	101.51	533.12	293	707	105.20	479.39	244	707	114.45
EE	758.24	82	942	219.14	760.57	553	897	102.39	759.38	82	942	172.48
Methane												
g/day	127.83	50.3	418	70.79	286.00	95.9	540	125.54	196.83	50.3	540	125.91
MJ/day	7.07	2.64	23.3	3.97	15.79	5.6	30.1	7.01	10.87	2.64	30.1	7.00
g/kg DM	18.6	6.61	34.1	5.87	20.25	12.2	28.9	4.07	19.32	6.61	34.1	5.23
g/kg OM	19.99	6.8	39.9	6.56	22.80	12.5	42.3	5.11	21.22	6.8	42.3	6.13
MJ/kg DM	1.03	0.37	1.9	0.32	1.12	0.68	1.58	0.22	1.07	0.37	1.9	0.29
% of GE	5.81	2.2	11.5	1.99	6.42	2.86	10.86	1.35	6.08	2.2	11.5	1.77
% of DE	8.36	2.89	16.9	3.12	9.06	3.49	17.9	2.62	8.65	2.89	17.9	2.94
g/kg digestible DM	27.68	9.58	54.89	9.36	29.31	15.04	57.06	7.23	28.39	9.58	57.06	8.54
g/kg digestible OM	28.23	9.58	59	10.34	29.48	14.62	55.41	7.59	28.80	9.58	59	9.23

Min, minimum value in the database; Max, maximum value in the database; SD, standard deviation; ADF, acid detergent fiber; CP, crude protein; DE, digestible energy; DM, dry matter; EE, ether extract; GE, gross energy; ME, metabolizable energy; NDF, neutral detergent fiber; NFC, non-fibrous carbohydrate; OM, organic matter; OMDm, organic matter digestibility at maintenance level. ^a^ Feeding level as a multiple of maintenance metabolizable energy intake.

**Table 2:** List of published equations used to predict CH_4_ production from beef and dairy cows.

Source	Equation
Mills et al. (2003) [5]	Methane (MJ/day) = 5.93 + 0.92 × DMI (kd/day)
	Methane (MJ/day) = 56.27 − (56.27 + 0) × exp[−0.028 × DMI (kg/day)]
IPCC (2006) [16]	Methane (MJ/day) = 0.065 × GEI (MJ/day)
Ellis et al. (2007) [6]	Methane (MJ/day) = 3.272 + 0.736 × DMI (kd/day)
Yan et al. (2009) [7]	Methane (MJ/day) = 0.582 + 1.40 × DMI (kd/day)
Ramin and Huhtanen (2013) [8]	Methane (MJ/day) = 0.797 + 1.427 × DMI (kd/day) − 0.020 × DMI (kg/day)^2^
Patra (2015) [9]	Methane (MJ/day) = 35.21 − (35.21 + 0.25) × exp[−0.0354 × DMI (kg/day)]

## Data Availability

The data presented in this study are available on request from the corresponding author.
